# Indole-3- carbinol enhances sorafenib cytotoxicity in hepatocellular carcinoma cells: A mechanistic study

**DOI:** 10.1038/srep32733

**Published:** 2016-09-09

**Authors:** Mai M. Abdelmageed, Reem N. El-Naga, Ebtehal El-Demerdash, Mohamed M. Elmazar

**Affiliations:** 1 Department of Pharmacology and Toxicology, Faculty of Pharmacy, The British University in Egypt (BUE), Cairo, Egypt; 2 Department of Pharmacology and Toxicology, Faculty of Pharmacy, Ain Shams University, Cairo, Egypt; 3 Department of Pharmacology and Toxicology, Faculty of Pharmacy, Misr International University, Cairo, Egypt

## Abstract

Sorafenib is the only chemotherapeutic agent currently approved for unresectable hepatocellular carcinoma (HCC). However, poor response rates have been widely reported. Indole-3-carbinol (I3C) is a potential chemopreventive phytochemical. The present study aimed to explore the potential chemomodulatory effects of I3C on sorafenib in HCC cells as well as the possible underlying mechanisms. I3C exhibited a greater cytotoxicity in HepG2 cells compared to Huh-7 cells (p < 0.0001). Moreover, the co-treatment of HepG2 cells with I3C and sorafenib was more effective (p = 0.002). Accordingly, subsequent mechanistic studies were carried on HepG2 cells. The results show that the ability of I3C to enhance sorafenib cytotoxicity in HCC cells could be partially attributed to increasing the apoptotic activity and decreasing the angiogenic potentials. The combination had a negative effect on epithelial-mesenchymal transition (EMT). Increased NOX-1 expression was also observed which may indicate the involvement of NOX-1 in I3C chemomodulatory effects. Additionally, the combination induced cell cycle arrest at the G_0_/G_1_ phase. In conclusion, these findings provide evidence that I3C enhances sorafenib anti-cancer activity in HCC cells.

Hepatocellular carcinoma (HCC) is the fifth most prevalent malignancy in the world and the third major cause of cancer mortality^1^. HCC presents with high incidence rates in the developing countries in East Asia and sub-Saharan Africa where 80% of the cases occur^1^. Moreover, most HCC patients have poor prognosis and show resistance to chemotherapy^2^.

Sorafenib, an oral multikinase inhibitor, possesses potential activity against several receptor tyrosine kinases including vascular endothelial growth factor receptor (VEGFR) 1, 2 and 3, as well as, platelet-derived growth factor receptor-β (PDGFR-β)^3^. In 2008, the Sorafenib Hepatocellular Carcinoma Assessment Randomized Protocol (SHARP) trials introduced sorafenib as the standard treatment for patients with advanced HCC^4^. Despite the efficacy demonstrated by sorafenib in clinical trials, tumor response rates were modest suggesting the development of multiple drug resistance mechanisms^2^. The activation of the epidermal growth factor receptor (EGFR), overexpression of hypoxia inducible factor 1-α (HIF 1-α) and epithelial-mesenchymal transition (EMT) are major mechanisms reported to contribute to sorafenib resistance^5^,^6^,^7^. This assures the need for new agents improving the therapeutic outcome of sorafenib.

Recently, studies have shown that dietary phytochemicals might contribute to a reduced HCC risk^8^. Among these promising phytochemicals is indole-3-carbinol (I3C). I3C is a potential chemopreventive agent with multiple anti-tumor activities including apoptotic, anti-proliferative and anti-angiogenic activities^9^. It occurs naturally in cruciferous vegetables^10^. Wang *et al*.^11^ showed that the anti-angiogenic activities of I3C are attributed to the decreased production of VEGF, matrix metalloproteinases (MMPs) and nitric oxide (NO). I3C also modulates key signaling pathways such as phosphoinositide 3-kinase (PI3K)/Akt survival pathway^12^. Several clinical trials have investigated I3C for cancer treatment and prevention. Interestingly, I3C has been reported to be effective in clinical studies against precancerous lesions caused by human papillomavirus in cervix^13^ and larynx^14^ with no reported adverse events. Moreover, current clinical trials are investigating the potential utilization of natural supplements including I3C in eliminating cancer-influencing exogenous estrogens in patients with prostate, breast and uterine cancers^15^. An ongoing clinical trial is also evaluating the intake of cruciferous vegetables and associating their intake with histone deacetylase expression and histone status in patients undergoing scheduled screening colonoscopy for colon cancer^16^.

Indeed, the selective cytotoxic potential of I3C towards tumorigenic cells should be investigated in combination with chemotherapeutic agents^17^. Accordingly, the present study aimed to investigate the possible chemomodulatory effects of I3C on sorafenib in the human HCC cell lines. Also, the putative mechanisms underlying this modulation were explored in terms of their effects on apoptotic and angiogenic machineries as well as on tumor invasiveness. Light was shed on the contribution of NADPH oxidase 1 (NOX-1), a member of the NOX family of enzymes, in I3C modulatory effects as well as on clusterin which has been reported to be involved in HCC metastasis and chemoresistance^18^.

## Results

### I3C significantly enhanced the cytotoxicity of sorafenib on HepG2 cells

**Figure 1:**
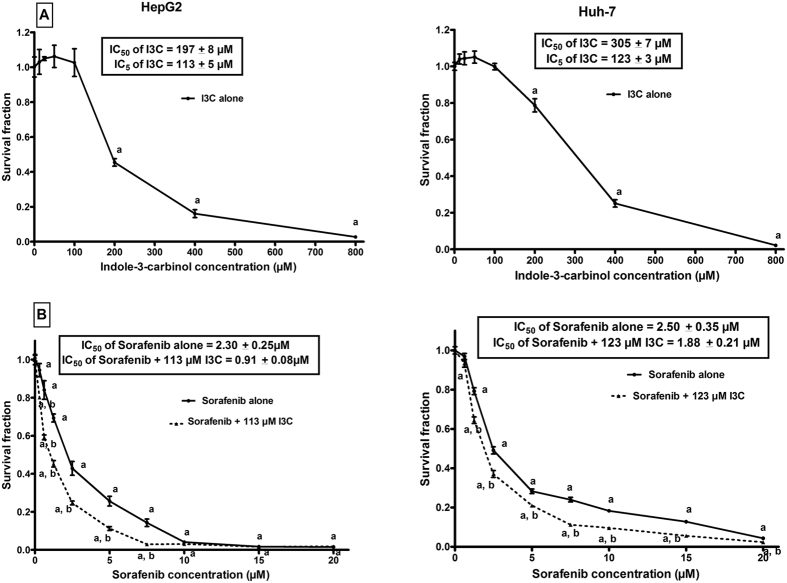
Cytotoxicity of I3C, sorafenib and their combination in HepG2 and Huh-7 cells. (**A**) Cytotoxicity of various concentrations of I3C in HepG2 and Huh-7 cell lines. Data are means ± SD of 3 independent experiments. ^a^p < 0.05: statistically significant when compared to the control value using ANOVA followed by Dunnett test as post-hoc test. (**B**) Cytotoxicity of various concentrations of sorafenib alone or in combination with I3C in HepG2 and Huh-7 cell lines. Data are means ± SD of 3 independent experiments. ^a^p < 0.05: statistically significant when compared to the control value using ANOVA followed by Dunnett test as post-hoc test. ^b^p < 0.05: statistically significant when compared to the corresponding group treated with sorafenib alone using unpaired t-test.

To elucidate the effect of I3C on sorafenib cytotoxicity, the concentration-survival curve of sorafenib on HepG2 and Huh7 cells was generated and compared to that obtained after co-treatment with I3C. Treatment of HepG2 and Huh-7 cells with different concentrations of sorafenib (0.31–20 μM) for 72 h significantly decreased the growth of cells in a concentration-dependent manner. From the fitted survival curves, the IC_50_ of sorafenib was calculated and was found to be 2.30 ± 0.25 μM and 2.50 ± 0.35 μM in HepG2 and Huh-7 cells, respectively. Also, the treatment of HepG2 and Huh-7 cells with varying concentrations of I3C (12.50–800 μM) for 72 h significantly inhibited the growth of cells in a concentration-dependent manner (Fig. 1A). The cytotoxicity of I3C was more significant (p < 0.0001) in HepG2 cells compared to Huh-7 cells. From the concentration-survival curve, the IC_5_ of I3C was determined and selected to study the modulatory effects of I3C on sorafenib. The combination of I3C with sorafenib significantly enhanced the sensitivity of cells to sorafenib cytotoxicity where the IC_50_ of sorafenib was markedly reduced to be 0.91 ± 0.8 μM (combination index (CI) = 0.97, i.e. an additive effect) and 1.88 ± 0.21 μM (CI = 1.15, i.e. an additive effect) in HepG2 and Huh-7 cells, respectively (Fig. 1B). The reduction in sorafenib IC_50_ after the co-treatment with I3C was significantly (p = 0.002) more pronounced in HepG2 cells (2.53- folds) compared to Huh-7 cells (1.33- folds) and hence, the subsequent DNA-ploidy flowcytometric analysis and mechanistic studies were performed on HepG2 cells.

### The combined treatment of I3C with sorafenib induced cell cycle arrest

**Figure 2:**
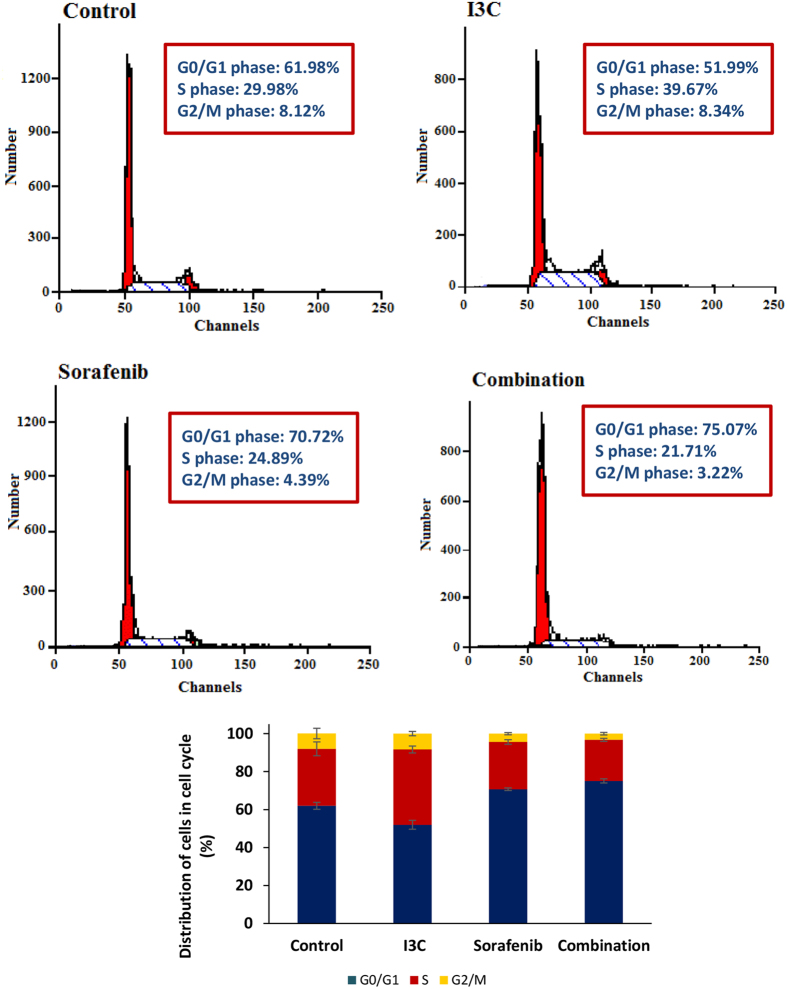
Cell cycle analysis of HepG2 cells after 72 h of treatment with 0.3% DMSO, 113 μM I3C, 2.30 μM sorafenib, and their simultaneous combination. The X-axis represents the channel number (relative DNA content/cell). The Y-axis represents the number of cells/channel. Data are means ± SD of 3 independent experiments.

To better understand the mechanism responsible for the potential chemomodulatory effects of I3C on sorafenib, the cell cycle distribution was evaluated using flowcytometric analysis. Treatment of HepG2 cells with I3C alone caused a significant S phase arrest of the cell cycle by 32.32% as compared to the untreated cells. On the other hand, sorafenib caused a significant G_0_/G_1_ phase arrest by 14.10% as compared to control cells (Fig. 2). Interestingly, it was found that sorafenib and I3C co-treatment caused a prominent G_0_/G_1_ phase block by 21.11% and 6.15%. This significant G_0_/G_1_ phase arrest occurred on the expense of S and G_2_/M phases.

### The combination of I3C and sorafenib significantly induced apoptosis

**Figure 3:**
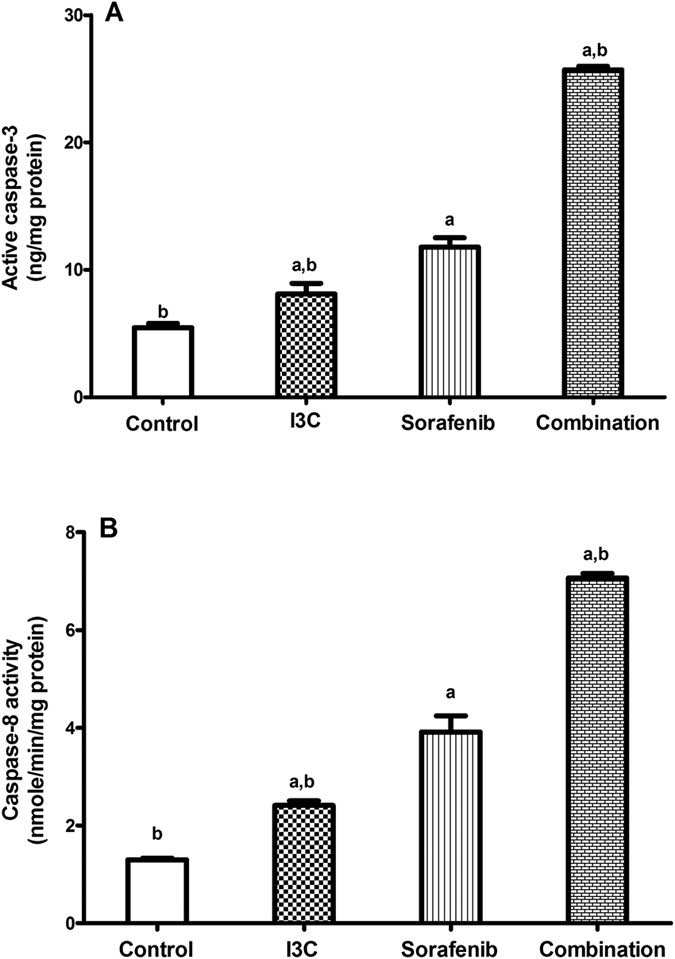
The effect of 113 μM I3C, 2.30 μM sorafenib and their combination on (**A**) caspase-3 and (**B**) caspase-8 activities. Each point is the mean ± SD of 3 independent experiments. a or b: Significantly different from the control or sorafenib group, respectively, p < 0.05 using ANOVA followed by Tukey–Kramer as post-hoc test.

To study the effect of I3C, sorafenib and their combination on the apoptotic machinery, the level of caspase-3 and caspase-8 activities were assessed in different treated groups. Figure 3A shows the changes in active caspase-3 levels following the treatment of cells with the appropriate drug concentrations. Active caspase-3 levels were increased significantly following treatment with I3C, sorafenib and their combination by 1.49, 2.17 and 4.72 folds, respectively as compared to the control values. Notably, the 2 drugs significantly increased the levels of active caspase-3 by 118.18% as compared to the sorafenib group. Figure 3B shows the effect of the different treatments on caspase-8 activity. I3C, sorafenib and their combination markedly enhanced caspase-8 activity reaching 1.87, 3.01 and 5.44 folds, respectively as compared to the control group. Again, the combination succeeded in activating the enzyme more than the individual treatments reaching 80.62% as compared to sorafenib-treated group.

### The combination significantly downregulated the levels of p-Akt, p-ERK and HIF-1α

**Figure 4:**
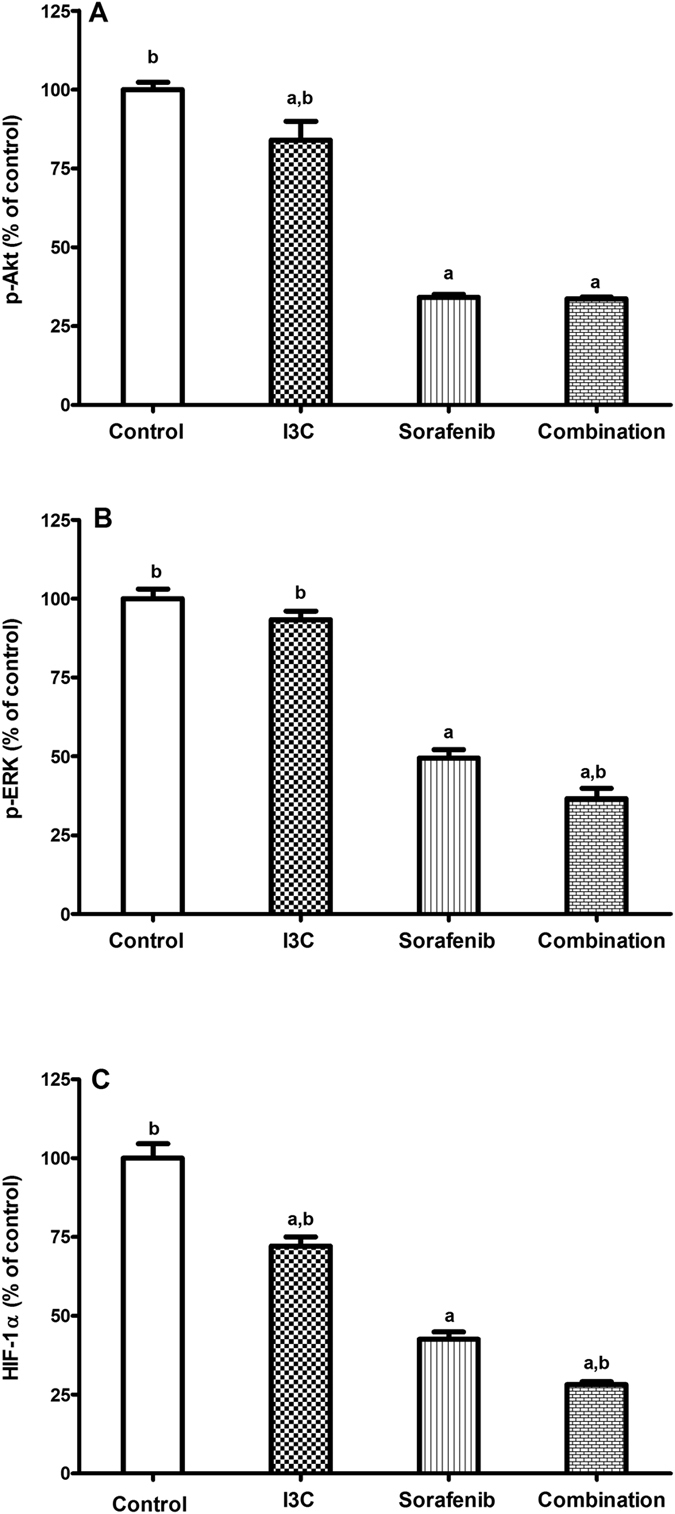
The effect of 113 μM I3C, 2.30 μM sorafenib and their combination on the levels of (**A**) p-Akt, (**B**) p-ERK and (**C**) HIF-1α. Data is given as percentage of control. Each point is the mean ± SD of 3 independent experiments. a or b: Significantly different from the control or sorafenib group, respectively, p < 0.05 using ANOVA followed by Tukey–Kramer as post-hoc test.

To study the effects of the different treatments on cell proliferation and angiogenesis the levels of p-Akt, p-ERK and HIF-1α were measured. Treatment of HepG2 cells with I3C and sorafenib significantly decreased the levels of p-Akt by 16.02% and 65.87%, respectively as compared to the control values. However, the reduction in p-Akt levels caused by the combination was insignificant as compared to the sorafenib only treated group (Fig. 4A). Figure 4B shows that the treatment of HepG2 cells by sorafenib markedly reduced the levels of p-ERK by 50.54% as compared to the control group. Although the downregulation in p-ERK levels caused by I3C was insignificant, the combination induced a significant reduction of p-ERK levels by 63.31% and 25.95% as compared to control and sorafenib only treated groups, respectively. I3C and sorafenib decreased the levels of HIF-1α by 27.86% and 57.44%, respectively as compared to control. Furthermore, treatment of HepG2 cells with both I3C and sorafenib showed a further significant decrease in HIF-1α levels by 33.79% as compared to the sorafenib only treated group (Fig. 4C).

### The combination significantly downregulated the levels of snail and clusterin

**Figure 5:**
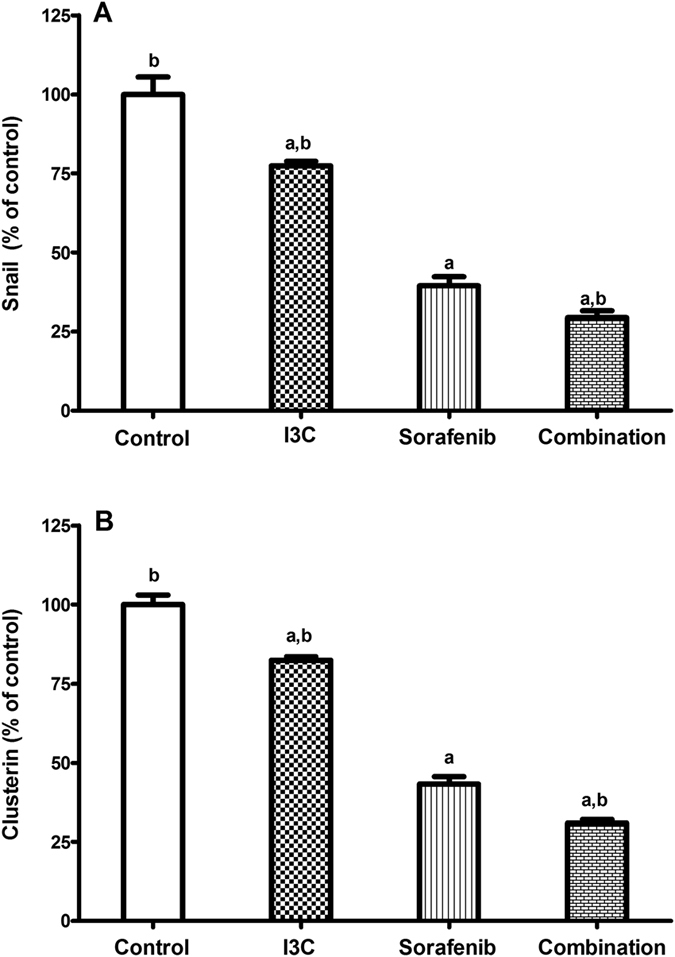
The effect of 113 μM I3C, 2.30 μM sorafenib and their combination on the levels of (**A**) snail, (**B**) clusterin. Data is given as percentage of control. Each point is the mean ± SD of 3 independent experiments. a or b: Significantly different from the control or sorafenib group, respectively, p < 0.05 using ANOVA followed by Tukey–Kramer as post-hoc test.

To further investigate the potential chemomodulatory effects of I3C on sorafenib, the levels of snail and clusterin were measured. Indeed, sorafenib caused 60.47% and 56.75% reduction in the levels of snail and clusterin, respectively as compared to the control. The combination further decreased the levels of snail and clusterin by 25.58% and 28.28% respectively as compared to the sorafenib only treated group (Fig. 5A,B).

### The combination significantly increased the expression of NOX-1

**Figure 6:**
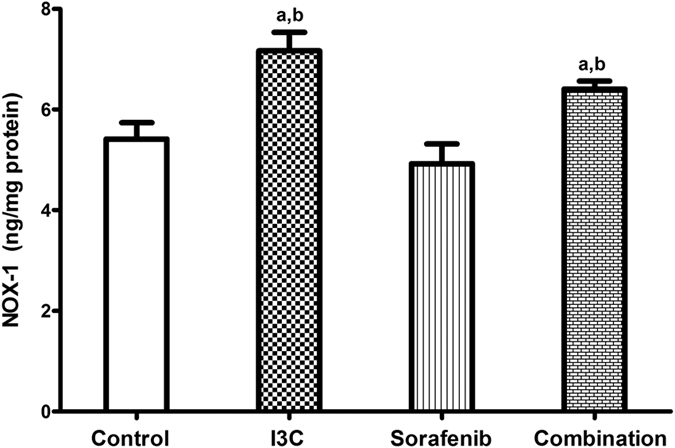
The effect of 113 μM I3C, 2.30 μM sorafenib and their combination on the levels of NOX-1. Each point is the mean ± SD of 3 independent experiments. a or b: Significantly different from the control or sorafenib group, respectively, p < 0.05 using ANOVA followed by Tukey–Kramer as post-hoc test.

The levels of NOX-1 were measured to assess the involvement of NADPH enzymes in I3C cytotoxic potentials (Fig. 6). Interestingly, I3C significantly increased the expression of NOX-1 by 1.33 folds as compared to the untreated cells whereas sorafenib failed to achieve any significant change in the levels of NOX-1 as compared to the control. In the combination regimen, the levels of NOX-1 were increased significantly by 1.18 and 1.30 folds as compared to control and sorafenib only treated groups, respectively (Fig. 6).

### The combination significantly decreased the gene expression of VEGF and EGFR and significantly increased the gene expression of E-cadherin

The effects of I3C, sorafenib and their combination on the gene expression of VEGF, EGFR and E-cadherin were assessed. In the mentioned concentration, I3C caused no significant change in the mRNA levels of VEGF or E-cadherin but significantly downregulated the expression of EGFR by 15.34% (Table 1). On the other hand, sorafenib significantly decreased the expression of VEGF and EGFR by 26% and 24% as compared to the control values. In addition, sorafenib significantly increased the levels of E-cadherin by 1.8 folds as compared to the control. In combination, the gene expression of VEGF and EGFR were decreased significantly by 64% and 36.5% respectively as compared to the control values. It is worth mentioning that while the mRNA levels of the VEGF were significantly decreased in the combination compared to the sorafenib group, the combination regimen failed to achieve any significant decrease in the expression of the EGFR as compared to sorafenib only treated cells. Indeed, the expression of E-cadherin was significantly augmented by the combination reaching 3.46 and 1.92 folds as compared to the control and sorafenib groups, respectively.

## Discussion

Sorafenib is the first and only pharmacological agent clinically approved for the treatment of advanced HCC^19^. Since several studies reported modest response to sorafenib therapy, the present study aimed to investigate whether I3C can modulate sorafenib cytotoxicity. Interestingly, it was found that co-treatment of HepG2 and Huh-7 cells with I3C and sorafenib significantly enhanced sorafenib cytotoxicity where the IC_50_ of sorafenib was reduced by 60.43% and 24.80%, respectively. To gain further insight into the putative mechanisms underlying that modulation, the effects of the combination on the apoptotic and angiogenic machineries as well as on tumor invasiveness were studied in HepG2 cells.

Initially, flowcytometric DNA ploidy analysis was performed to determine the cell cycle distribution in the different treated groups. It was found that I3C caused a preferential significant S-phase block of HepG2 cells on the expense of G_0_/G_1_ phase. While some studies have reported a prominent G_1_ cell cycle arrest to be induced by I3C^20^, others have reported a concomitant S-phase block^21^. Taylor-Harding *et al*.^22^ showed that the treatment of human ovarian carcinoma cells, OVCAR3, with I3C induced a concentration-dependent decrease in the percentage of cells in the G_1_ phase, whereas OVCAR5 cells only showed G_1_ phase arrest at maximal concentration of I3C up to 675 μM and arrested the cells at the S phase at a lower concentration of 450 μM. This may help to explain our finding by adopting the idea that I3C mediated cell cycle arrest might be concentration and cell-type dependent. On the other hand, sorafenib induced a significant cell cycle arrest at the G_0_/G_1_ phase. This result is in accordance with another study conducted on the same cell line at nearly the same concentration^23^. Interestingly, the co-treatment of HepG2 cells with I3C and sorafenib induced a significant accumulation of HepG2 cells in G_0_/G_1_ phase which suggests that I3C induced G_1_ phase arrest in HepG2 cells needed to be potentiated by another agent, sorafenib here, to become pronounced.

Accordingly, the effects of the different treatment groups on the apoptotic machinery were assessed by measuring the levels of caspase-3 as well as caspase-8 activities. It was found that the results of both caspases were consistent with each other. I3C *per se* significantly elevated the levels of caspase-3 along with caspase-8 activities. The combined treatment of I3C with sorafenib induced a more prominent elevation in the levels of caspase-3 as well as caspase-8 activities when compared to the respective sorafenib only treated group. Previously, sorafenib has been shown to promote the activation of caspase-8 followed by caspase-3 activation in HCC cells^24^. Moreover, these findings are in line with those found by Kim *et al*.^25^ showing that I3C decreases the expression of Bcl-2 and subsequently activate caspase-8 and caspase-3 in human melanoma cells. Furthermore, I3C was found to sensitize HepG2 cells to TRAIL-induced apoptosis^26^. It is therefore not surprising that the combination of both agents increased caspase activity, indicating the activation of the apoptotic machinery. Also, since caspase-8 is a key initiator caspase of death receptors mediated apoptosis, it will not be surprising if the extrinsic pathway of apoptosis was activated by the combination. However, we cannot claim that there is a preference towards the activation of the extrinsic pathway to be induced by the combination without the involvement of the intrinsic pathway. In fact, the two apoptotic pathways, the extrinsic and the intrinsic, work in conjugation with each other, they go in parallel and usually the extrinsic pathway activates the intrinsic pathway to help it exerting its action^27^,^28^.

The highly vascularized nature of HCC shows the importance of anti-angiogenic treatments which hinder the formation of blood vessels in cancerous tissues. Additionally, a clinical study conducted by Huang *et al*.^29^ reported that HCC neovascularization is strongly associated with HIF-1α overexpression, possibly through VEGF regulation. To investigate the possible modulatory effects exerted by I3C on sorafenib anti-angiogenic potentials, the protein levels and mRNA expression of some key regulators of cell proliferation and angiogenesis were assessed.

The EGFR is a receptor tyrosine kinase which regulates multiple processes in tumorigenesis including cell survival and angiogenesis^30^. While some studies have shown that anti-EGFR therapies might be clinically useful in overcoming sorafenib resistance^5^, others reported a diminished effect of these agents in combination with sorafenib^31^. In accordance with our results, Chinni *et al*.^32^ showed that I3C could suppress the EGFR levels with a concomitant inhibition of Akt phosphorylation in PC-3 cells. However, the diminished effect of the combination on the expression of EGFR and p-Akt levels compared to the respective sorafenib group suggests that the interactions between I3C and sorafenib are independent of EGFR/Akt signaling and that other molecules are involved in I3C mediated sensitization of HepG2 cells to sorafenib.

The ERK signaling pathway has been demonstrated to mediate a variety of functions including cancer cell proliferation^33^. I3C has been shown to inhibit hepatic stellate cell proliferation through the inhibition of p38 MAPK phosphorylation while it had no effect on p-ERK^34^. Nevertheless, another study showed that I3C suppresses invasion and MMP-2 expression in breast cancer MCF-7 cells through the inhibition of ERK signaling pathway with no effect on Akt activity^35^. Hence, I3C effects on different signaling pathways are highly dependent on cell type. Regarding the anti-angiogenic activities of I3C, previous studies have reported that I3C could downregulate VEGF secretion and suppress angiogenesis in endothelial cells activated by macrophages^11^ and also in phorbol myristate acetate (PMA)-activated endothelial cells^36^. Thus, we might conclude that the anti-angiogenic modulatory effects exerted by I3C on sorafenib in HepG2 cells are partially attributed to the downregulation of HIF-1α and its downstream molecule, VEGF, through p-ERK signaling rather than p-Akt.

Epithelial-mesenchymal transition (EMT) refers to the transient shift of cells from a polarized epithelial state to a mesenchymal one of high motility and invasiveness^37^. This biological process plays a serious role in cancer metastasis and invasion and has been widely studied in HCC^38^. Malenstein and his co-workers^39^ showed that the treatment of HepG2 cells with sorafenib for a long time can promote sorafenib resistance along with EMT development and increased invasiveness. Essentially, EMT induction is detected by the suppression of E-cadherin expression *via* several transcription factors as snail and slug^40^,^41^. Thus, to assess the ability of I3C to suppress the progression of EMT in combination with sorafenib, the mRNA expressions of E-cadherin as well as the protein levels of snail were measured. Moreover, clusterin was assessed in the different treatment groups. HepG2 cells co-treated with I3C and sorafenib showed a prominent upregulation in E-cadherin expression along with a significant downregulation in snail and clusterin levels. Previously, I3C has been reported to suppress EMT and migration of breast cancer cells through the repression of focal adhesion kinase (FAK) leading to decreased MMPs activity and upregulation of E-cadherin expression^42^. Here, we denote that I3C mediated repression of EMT involves the downregulation of snail and clusterin leading to increased E-cadherin expression. Several studies have reported the upregulation of clusterin expression in several cancers^43^ including HCC^44^. Wang *et al*.^18^ reported the contribution of clusterin to EMT and HCC metastasis. Moreover, clusterin has been shown to increase HCC invasiveness through decreased E-cadherin expression and increased MMP-2 expression^45^. It is worth mentioning that clusterin inhibition sensitizes human renal cell carcinoma to sorafenib^46^.

NADPH oxidases belong to a family of enzymes which generate reactive oxygen species (ROS) and play a significant role in several physiological and pathological conditions^47^. Previously, ROS has been correlated with the adhesion and migration of cells^48^ and NOX-1 has been reported to be a cellular source for these free radicals^49^. Some of the tumorigenic potentials of NOX-1 were attributed to its protective actions against the transforming growth factor-β (TGF-β) proapoptotic signals^50^ and its contribution to autocrine cell growth *via* EGFR^51^. On the contrary, the expression of NOX-1 was augmented in the well-differentiated adenocarcinoma cells and was downregulated in the poorly-differentiated cells highlightening that NOX-1 expression is not linked with the degree of malignancy^52^. Moreover, NOX-1 expression was not significantly higher in colon cancer compared to normal colon tissues^53^ implying that NOX-1 might play a role in the differentiation of colon epithelial cells rather than carcinogenesis^54^. Interestingly, ROS-mediated mechanisms might represent a new therapeutic approach for cancer cell targeting^55^. Additionally, oxidative stress and ROS production has been reported to induce apoptosis^56^. By the same token, NOX-mediated generation of ROS has been shown to enhance the death of HepG2 cells^57^. Thus, to study the role of NADPH oxidase enzymes in I3C mediated sensitization of HepG2 cells to sorafenib, the level of NOX-1 expression was determined. Surprisingly, the levels of NOX-1 expression were significantly augmented in I3C and the combination groups. A result which may support that NOX-1 production may contribute to I3C anticancer modulatory activities. In accordance with our results, the anti-tumour activities of sorafenib have been greatly enhanced with other agents increasing ROS production^58^. In this context, we may suggest the possible role of NOX-1 in improving the therapeutic outcomes of sorafenib therapy which was not stated before.

Finally, an interesting study conducted by Elmore *et al*.^59^ examined the toxicity of I3C in several normal human epithelial cells including human hepatocytes. The toxic concentrations of I3C in normal hepatocytes which inhibited cell growth, mitochondrial function and albumin synthesis were 589.9 μM, >1019 μM and 245.7 μM, respectively. It is intriguing that these concentrations are considerably higher than the concentration of I3C used in the present study against HCC cell lines which might shed some light on safety to the normal surrounding tissue and hence, support the selective toxicity of I3C to HCC cells. I3C was well-tolerated in clinical trials^13^,^14^. Moreover, I3C supplements were reported to be taken for approximately 7 years with no reported side effects, suggesting long-term safety of this natural product^10^. Indeed, several studies have also reported the hepatoprotective effects of I3C against hepatotoxic agents^60^ including anti-tumor drugs^61^. Taking into the account the previously reported hepatotoxicity encountered when sorafenib is used in combination with antiviral agents for the treatment of viral hepatitis related HCC^62^, the current study provides a hopeful solution.

In conclusion, this was the first study to show that I3C, at a subtoxic concentration, enhances the cytotoxic activities of sorafenib in HCC cells. This could be, at least, partially attributed to activating the apoptotic machinery, increasing the anti-angiogenic potentials, attenuating EMT as well as increasing NOX-1 expression. Taken together the reported safety of I3C in clinical trials, the present study offers a rationale for a new therapeutic combination for the treatment of HCC. Nevertheless, further *in vitro* and *in vivo* studies, as well as, rationally designed clinical trials are required to fully elucidate the potential therapeutic outcomes of this combination.

## Materials and Methods

### Drugs and chemicals

Sorafenib, p- Toluenesulfonate Salt, was purchased from LC Laboratories (Woburn, MA, USA). I3C was purchased from Sigma-Aldrich (St. Louis, MO, USA). DMSO and other chemicals were purchased from Sigma-Aldrich (St. Louis, MO, USA). Fetal bovine sera, media and other cell culture materials were purchased from Gibco Life Technologies Ltd. (Grand Island, NY, USA).

### Cell culture

The human HCC cell lines, HepG2 and Huh-7, were obtained from the Vaccera (Giza, Egypt). HepG2 cells were cultured in RPMI-1640 medium supplemented with 1% penicillin-streptomycin antibiotic and 10% (v/v) heat-inactivated fetal bovine serum. Huh-7 were maintained in DMEM supplemented as mentioned above. The cell lines were incubated at 37 °C in a humidified 5% CO_2_-95% air. In the mechanistic studies, HepG2 cells were divided into four different treatment groups; the first group (control) was incubated with 0.3% DMSO, the second group was treated with 113 μM I3C, the third group was treated with 2.30 μM sorafenib and finally the fourth group was co-treated with 113 μM I3C and 2.30 μM sorafenib. All treatments were carried out for 72 h.

### Cytotoxicity assay and data analysis

HepG2 and Huh-7 cells were seeded at a density of 4-5 × 10^3 ^cells/well in 96-well plates. Cells were incubated for 24 h at 37 °C with 5% CO_2_. Initially, cells were exposed to varying concentrations of either sorafenib or I3C for 72 h. Sorafenib was used at concentrations ranging from 0.31 to 20 μM whereas I3C was used at concentrations ranging from 12.50 to 800 μM. Wells serving as control were treated with 0.30% DMSO, the vehicle, only. At the end of drug exposure, cytotoxicity was assessed using Sulphorhodamine-B (SRB) method as previously reported^63^. The absorbance of each well was measured at 545 nm with an ELISA microplate reader (ChroMate-4300, FL, USA). Concentration-survival curves were generated, and the drug concentrations at which 50% of cell growth were inhibited (IC_50_), were calculated (Graph Pad, Prism software, version 5). From fitted survival curves, the concentration of I3C that inhibited only 5% of cells was determined. This concentration was used for studying the modulatory effects of I3C on sorafenib in HepG2 cells. The extent of interaction between I3C and sorafenib in combination was evaluated by applying the isobologram equation (Eq. 1):


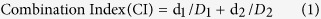


where d_1_ and d_2_ are the corresponding concentrations of I3C and sorafenib used in combination to achieve a certain level of growth inhibition, while D_1_ and D_2_ are their concentrations capable of producing alone the same magnitude of growth inhibition. The effect of combination is said to be synergistic if CI < 0.8; antagonistic if CI > 1.2; and additive if CI ranges from 0.8–1.2^64^.

### Cell cycle analysis by flow cytometry

HepG2 cells were plated in T_75_ flasks at a density of 1 × 10^6 ^cells/flask in RPMI-1640 supplemented medium. One day later, when 30% confluent, cells were treated with the indicated concentrations of I3C and sorafenib for 72 h. Then, cells were trypsinized and washed twice with phosphate buffer saline (PBS). The cellular DNA was stained using CycleTEST^TM^ PLUS DNA Reagent Kit (BD Biosciences, San Jose, CA) according to manufacturer’s instructions. Cell cycle analysis was performed using the Becton-Dickinson FACScan flow cytometer (BD Biosciences, San Jose, CA) and the CELLQuest software (BD Biosciences, San Jose, CA). Data from at least 10,000 events were used in each analysis. The percentage of cell distribution in G_0_/G_1_, G_2_/M and S phases was displayed by the planimetry of the histogram.

### Determination of caspase-3 and caspase-8 activities

Active caspase-3 was determined quantitatively using active caspase-3 ELISA kit (R&D Systems, Minneapolis, MN). HepG2 cells were seeded at a density of 5 × 10^4 ^cells/well in 6-well plates and treated with the appropriate drug concentrations. Following drug exposure, cells were incubated with biotin-ZVKD-fmk inhibitor for 1 h to label the active caspases. After incubation, cell extracts were prepared according to the manufacturer’s instructions. Then, 100 μl of samples and standards were added to active caspase-3 microplate pre-coated with caspase-3 antibody and incubated for 2 h at room temperature. Wells were washed and 100 μl of active caspase-3 conjugate were added and incubated for 1 h. Wells were washed again and 100 μl of substrate solution were added to wells and incubated for 30 min in the dark. The reaction was terminated by the addition of 100 μl of stop solution followed by measuring the optical density of the wells at 450 nm using correction wavelength set at 540 nm.

Caspase-8 activity was determined using caspase-8 colorimetric assay kit (Sigma-Aldrich, St. Louis, MO, USA). According to the manufacturer’s protocol, cells were harvested then lysed with 1 × lysis buffer. The reaction required 50 μl of cell lysate, 40 μl 1× Assay buffer and 10 μl of caspase-8 colorimetric substrate (Ac-IETD-p-nitroaniline). The optical density of the released p-nitroaniline was measured spectrophotometrically at 405 nm using ELISA microplate reader.

### Assessment of p-Akt, p-ERK and HIF-1α levels

The level of p-Akt (Ser473) was determined using the RayBio^®^ phosphor-Akt (Ser473) ELISA kit (RayBiotech, Inc., Norcross, GA, USA). At the end of treatment, approximately 4–5 × 10^6 ^cells were harvested and suspended in the 1× cell lysate buffer. According to the manufacturer’s manual, 100 μl of samples and positive control were added to 96-well microplate pre-coated with anti-pan Akt antibody and incubated for 2.5 h at room temperature. After washing, 100 μl of 1× detection antibody were added and allowed to incubate for 1 h at room temperature then washed. Then, 100 μl of prepared 1x HRP-Streptavidin solution were added to be incubated for another 1h and then washed. Finally, 100 μl of TMB Substrate were added to wells and incubated for 30 min in the dark followed by the addition of 50 μl stop solution. The optical density was measured immediately at 450 nm.

P-ERK and HIF-1α levels were assessed in cell culture supernatants using human HIF-1α and p-ERK ELISA kits (Abnova, Taipei, Taiwan). Both kits followed the same protocol where cell culture supernatants were collected by centrifugation. Samples and standards were added to the appropriate wells pre-coated with specific antibodies and kept for 2 h. Detection reagents A and B and the substrate were added and incubated according to the manufacturer’s instructions. The stop solution was added and the plates were measured specrophotometrically at 450 nm.

### Measurement of snail and clusterin levels

Human snail homolog 1 (SNAI1) was measured quantitatively using human SNAI1 ELISA kit (Cloud-Clone Corp., Houston, TX) while human clusterin (CLU) was determined using human clusterin ELISA kit (Kamiya Biomedical, Seattle, WA). The procedure was carried out according to the manufacturer’s protocol.

### Assessment of NOX-1 expression

ELISA kit for NOX1 (Cloud-Clone Corp., Houston, TX) was used to determine the level of NOX-1 expression in different treated groups. The procedure was carried out according to the manufacturer’s recommendations.

### Assessment of protein content

Protein content in cell lysates, cell extracts and culture supernatants was carried out using Bradford reagent (Sigma-Aldrich, St. Louis, MO, USA), where bovine serum albumin was used as a standard.

### Quantitative real-time polymerase chain reaction (qPCR) to analyze the gene expression of VEGF, EGFR and E-cadherin

Total RNA was extracted from cell cultures using GeneJET^™^ RNA Purification Kit (Fermentas, Life Sciences, Thermo Fisher). cDNA was synthesized using REVERTA kit (AmpliSens^®^, Russia) according to the manufacturers‘ recommendations. Real-time PCR was performed using specific primers for human VEGF, EGFR and E-cadherin (Table 2) and the Power SYBR^®^ Green PCR Master Mix (Applied biosystems, Warrington, UK). GADPH expression was used as the internal control. The values of the control groups were normalized to 1. Cycling conditions were 50 °C for 2 min, 95 °C for 10 min, followed by 40 cycles of 95 °C for 15 s, 60 °C for 30 s, 72 °C for 30 s followed by a final 10-min extension at 72 °C.

### Statistical analysis

All analyses were performed using GraphPad InStat software, version 3.05 (GraphPad Software, La Jolla, CA). Graphs were sketched using GraphPad Prism software, version 5.00 (GraphPad Software, La Jolla, CA). Data are presented as mean ± SD. Multiple comparisons were performed using one way analysis of variance (ANOVA) followed by either Dunnett test or Tukey-Kramer for post hoc analysis, as appropriate. Individual groups were compared using the unpaired Student’s t-test. The level of significance was set at p < 0.05.

## Additional Information

**How to cite this article**: Abdelmageed, M. M. *et al*. Indole-3- carbinol enhances sorafenib cytotoxicity in hepatocellular carcinoma cells: A mechanistic study. *Sci. Rep.* **6**, 32733; doi: 10.1038/srep32733 (2016).

## Figures and Tables

**Table 1:** The effects of I3C, sorafenib and their combination on the gene expression of VEGF, EGFR and E-cadherin.

Groups	VEGF	EGFR	E-cadherin
Control	1 ± 0.00^b^	1 ± 0.00^b^	1 ± 0.00^b^
I3C	0.86 ± 0.12	0.85 ± 0.03^a^	1.18 ± 0.20^b^
Sorafenib	0.74 ± 0.06^a^	0.76 ± 0.11^a^	1.81 ± 0.25^a^
Combination	0.36 ± 0.04a,b	0.63 ± 0.00^a^	3.46 ± 0.14^a,b^

Data is given as mean  ±  SD of 3 independent experiments. a or b: Significantly different from the control or sorafenib group, respectively, p < 0.05 using ANOVA followed by Tukey–Kramer as post-hoc test.

**Table 2:** Primers used in quantitative real time PCR.

Genes	Sense primer (5′–3′)	Antisense primer (5′–3′)
VEGF	AGGAGGGCAGAATCATCACG	TATGTGCTGGCCTTGGTGAG
EGFR	CAGCGCTACCTTGTCATTCAG	TCATACTATCCTCGGTGGTCA
E-cadherin	CTCCAGCTTGGGTGAAAGAG	GGGCTTTTACACTTGGCTGA
GADPH	GTGAAGGTCGGAGTCAACG	GGTGAAGACGCCAGTGGACTC

VEGF, vascular endothelial growth factor; EGFR, epidermal growth factor receptor; E-cadherin, epithelial cadherin; GADPH, glyceraldehyde phosphate dehydrogenase.
